# Different Modulation of Common Motor Information in Rat Primary and Secondary Motor Cortices

**DOI:** 10.1371/journal.pone.0098662

**Published:** 2014-06-03

**Authors:** Akiko Saiki, Rie Kimura, Toshikazu Samura, Yoko Fujiwara-Tsukamoto, Yutaka Sakai, Yoshikazu Isomura

**Affiliations:** 1 Brain Science Institute, Tamagawa University, Machida, Tokyo, Japan; 2 Graduate School of Brain Sciences, Tamagawa University, Machida, Tokyo, Japan; 3 JST CREST, Chiyoda-ku, Tokyo, Japan; 4 Department of Applied Molecular Bioscience, Graduate School of Medicine, Yamaguchi University, Ube, Yamaguchi, Japan; 5 Laboratory of Neural Circuitry, Graduate School of Brain Science, Doshisha University, Kizugawa, Kyoto, Japan; University of Alberta, Canada

## Abstract

Rodents have primary and secondary motor cortices that are involved in the execution of voluntary movements via their direct and parallel projections to the spinal cord. However, it is unclear whether the rodent secondary motor cortex has any motor function distinct from the primary motor cortex to properly control voluntary movements. In the present study, we quantitatively examined neuronal activity in the caudal forelimb area (CFA) of the primary motor cortex and rostral forelimb area (RFA) of the secondary motor cortex in head-fixed rats performing forelimb movements (pushing, holding, and pulling a lever). We found virtually no major differences between CFA and RFA neurons, regardless of neuron subtypes, not only in their basal spiking properties but also in the time-course, amplitude, and direction preference of their functional activation for simple forelimb movements. However, the RFA neurons, as compared with the CFA neurons, showed obviously a greater susceptibility of their functional activation to an alteration in a behavioral situation, a 'rewarding' response that leads to reward *or* a 'consummatory' response that follows reward water, which might be accompanied by some internal adaptations without affecting the motor outputs. Our results suggest that, although the CFA and RFA neurons commonly process fundamental motor information to properly control forelimb movements, the RFA neurons may be functionally differentiated to integrate motor information with internal state information for an adaptation to goal-directed behaviors.

## Introduction

Voluntary movements are controlled by the frontal part of the cerebral cortex in mammals. For example, primates have at least four frontal motor cortices with different motor functions in each hemisphere, namely, the primary motor cortex, supplementary motor area (SMA), premotor area (PM), and cingulate motor area (CMA) [1]. The primary motor cortex plays the most critical role in motor execution itself [2], [3]. The SMA and PM differentially contribute to versatile motor functions such as motor preparation, initiation, sequence and suppression [4]–[8], while the CMA is characteristically involved in motivational motor selection [9]–[11].

Rodents are known to cleverly perform voluntary movements [12]. So far, researchers have identified two distinct motor cortices in rodents, the primary and secondary motor cortices (M1 and M2, according to a standard brain atlas [13]). These motor cortices, mapped somatotopically by microstimulation [14]–[24], have reciprocal connections [25]–[27] as well as direct and parallel projections to the spinal cord [25], [28]. The motor cortices are activated during skilled voluntary movements with forelimbs [23], [29], [30], but it is not clear whether the rodent secondary motor cortex has differentiated motor function as seen in the primate SMA, PM, and CMA. To date, there is no evidence of any special function of the secondary motor cortex despite current expectation. However, some people do regard the lateral and medial parts of the agranular cortex (AGl and AGm) as primary and secondary motor cortices in rodents, respectively. In particular, the AGm is thought to participate not only in fundamental motor functions [31]–[34] but also in higher-order cognitive/motor functions including conditional response [35], action sequence chunking [36], and value-based action selection [37]. Yet the AGl and AGm, which are broad zones defined cytoarchitecturally, are not actually equivalent to genuine primary and secondary motor cortices, respectively [14], [21], [38] (see also Discussion). Therefore, it still remains unclear whether the rodent secondary motor cortex has differentiated motor function as compared with the primary motor cortex.

To address this issue, we focused on the forelimb areas of the rat primary and secondary motor cortices, which were identified by microstimulation as caudal and rostral forelimb areas (CFA and RFA [25]), respectively. In the CFA and RFA, we analyzed neuronal activity for quantitative comparisons with respect to basal spiking properties and functional activations during skilled forelimb movements. Furthermore, we examined the possible modulation of neuronal activity in these forelimb areas during similar forelimb movements in different behavioral situations (a rewarding response that leads to reward and a consummatory response that follows reward water).

## Materials and Methods

### Animal preparation

All experiments were carried out in accordance with the animal experiment protocol approved by Tamagawa University Animal Care and Use Committee (H22–32; 2010–2013). All surgery was performed under isoflurane anesthesia, and all efforts were made to minimize suffering. The experimental procedures that we used here were established in our previous studies [39]–[41]. Adult male rats (150–250 g; *N* = 37 Long-Evans, Institute for Animal Reproduction, Japan; *N* = 10 Wistar, Japan SLC, Japan; we note that no differences between the two strains were found in our study) were kept in their home cage under an inverted light schedule (lights off at 9 a.m. and lights on at 9 p.m.). Prior to the experiments, these rats were briefly handled by an experimenter (10–15 min, twice). Under 2.0–2.5% isoflurane anesthesia (Univentor 400 anesthesia unit, Univentor, Malta), the rats had head-attachments (Narishige, Japan) surgically attached to their skulls with tiny anchor screws (stainless steel, M1, 2 mm long) and dental resin cement (Super-Bond C & B, Sun Medical, Japan; Panavia F2.0, Kuraray Medical, Japan; Unifast II, GC Corporation, Japan). Their body temperatures were maintained at 37°C by an animal warmer (BWT-100, Bio Research Center, Japan) during isoflurane anesthesia. For electrophysiological recordings, two Teflon-coated silver wire electrodes (A-M systems, USA; 180 µm in diameter each) were implanted above the cerebellum as a reference and a ground. In some experiments, twisted Teflon-coated silver wire electrodes were implanted into the right upper forelimb (near the *biceps brachii*) to measure its electromyogram (EMG) activity. After recovery from the surgery (2–3 days later), the rats were deprived of drinking water in their home cage, where food was available *ad libitum*. Instead, they were able to obtain sufficient water as a reward for their daily task performance in the laboratory (within one week; >5–10 ml/100 g body weight a day). When necessary, an agar block (containing 15 ml water) was given to the rats in the home-cage to maintain over 80% of their original body weight (cf. [42] for water control).

### Behavioral tasks

**Figure 1:**
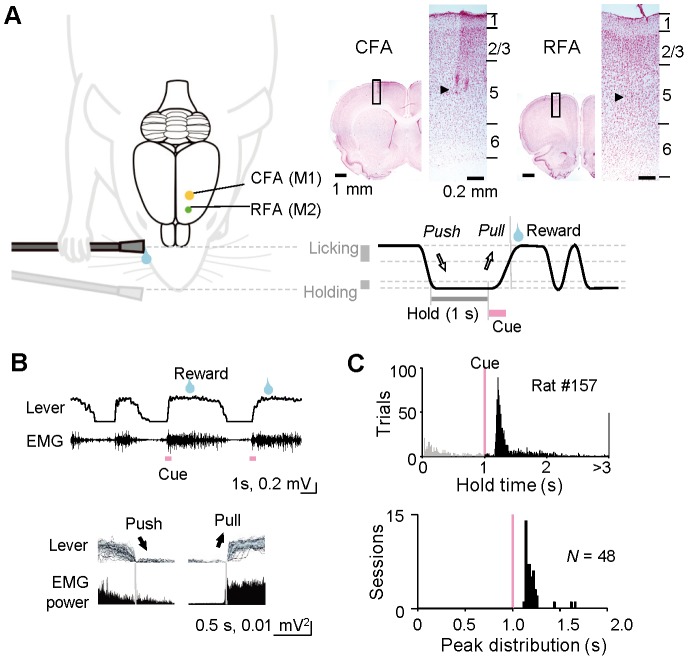
Behavioral task performance. **A**) A schematic of the forelimb movement task and recording sites for the two motor cortices. Rats held (for 1 s) and pulled a spout-lever to acquire reward water in a head-fixed condition. Multineuronal activity was recorded from the caudal and rostral forelimb areas (CFA and RFA) [primary and secondary motor cortices (M1 and M2), respectively] during task performance. *Upper right*: electrode tracks (arrowheads; layer 5) for the CFA and RFA recordings in Nissl-stained sections. See Materials and Methods for details. **B**) Lever trajectory and electromyogram (EMG) activity in right forelimb. *Top*: lever and EMG traces for several trials. *Bottom*: averaged EMG power aligned with the end of push or the onset of pull movements (vertical lines). **C**) Behavioral task performance. *Top*: hold time until the lever pull onset in response to cue tone presentation after the hold period (1 s) in a rat. Black and gray colors indicate correct and error trial responses, respectively. *Bottom*: peak distribution of hold time in all of the rats.

As established previously [40], we first trained the rats to perform a simple forelimb movement task (Fig. 1A), in which they had to manipulate a ''spout-lever'' with their right forelimb in a head-fixed condition. They spontaneously started each trial of this task by pushing the spout-lever forward and holding it for a short period (''hold period'') with the right forelimb. The hold period was extended from 0 ms up to 1,000 ms (final) in a step-by-step manner according to the total number of success trials. After the hold period was completed, a cue sound was briefly presented to them (10 kHz pure tone for 300 ms). If they pulled the spout-lever toward their mouth (holding position, 0–3 mm; licking position, 6–9 mm from the front end) in response to the cue presentation, then they were allowed to lick the spout-lever to drink 0.1% saccharin water (5 or 10 µl) as a reward. The reward was accurately dispensed from the tip of spout-lever by a micropump with a 200–800 ms delay (100 ms steps at random). The reward delivery period was followed by a short inter-trial interval (200–800 ms). Unless they held the spout-lever throughout the hold period, or unless they pulled it correctly within 5,300 ms (or 500 ms for Go trials in Go/No-go discrimination) after the cue onset, the rats were not rewarded (error trial) and had another attempt after the inter-trial interval. The rats typically learned the forelimb movement task within three days (2–5 hours a day) very efficiently using our automatic multi-rat task-training system (O'hara & Co., Ltd., Japan). Once the rats completed the operant learning of the forelimb movement task, they underwent a second surgery under anesthesia, and a tiny hole (1.0 to 1.5 mm in diameter) was made in the skull and dura mater above the left CFA (1.0±1.0 mm anterior, 2.5±1.0 mm lateral from bregma; mostly in the center of this area) or RFA (3.5±0.2 mm anterior, 2.4±0.2 mm lateral from bregma). These coordinates were determined by intracortical microstimulation (ICMS; −50 to −100 µA, 50 pulses at 100 Hz) to evoke reliable movement from the contralateral forelimb in our preliminary experiments (data not shown). In some cases, it was confirmed by the ICMS after a recording experiment (8–10 rats in each area). The hole was covered with silicon sealant (DentSilicone-V, Shofu, Japan). On the following day, they were transferred to a single behavioral experiment system (O'hara & Co., Ltd.) for final behavioral and electrophysiological experiments.

In the later part of this study, twenty-four rats were subjected to an additional behavioral experiment, in which they would perform similar forelimb movements in different behavioral situations using a Go/No-go response task [40]. The Go/No-go response task consisted of Go trials and No-go trials presented pseudo-randomly in a 1∶1 ratio. In the Go trials, the rats had to quickly pull the spout-lever less than 500 ms after the onset of the original (Go) cue (10 kHz for 300 ms) to acquire the reward. For convenience, we label the pull movement in Go trials as ''intentional pull''. In the No-go trials, the rats had to keep holding the spout-lever for at least 800 ms after the onset of the extension (No-go) cue (4 kHz for 300 ms). The reward was delivered 200–800 ms after a correct response for No-go trials as well as for Go trials symmetrically. Consequently, they licked the already-earned reward by pulling the spout-lever *after* a completion of correct No-go responses (''incidental pull''); the incidental pull would be seen each time a No-go response was completed. The intentional pull differed from the incidental pull in that a subject was operantly rewarded by the former, but not by the latter. In other words, we can consider the former and the latter as a 'rewarding' (operant) response (the response leads to reward) and a 'consummatory' response (water leads to the response), respectively. Thus, the incidental pull was neither an operant Go response nor a No-go response, but rather a kind of consummatory behavior. Hence, the rats would likely need more effortful information processing for a correct pull movement in Go than in No-go trials, whereas they would expect their reward acquisition more or less in both trial-types. If they failed to respond correctly to a new trial, the rats had to retry the same trial-type after the inter-trial interval until it was successfully cleared. In the course of such Go and No-go behaviors, we obtained sufficient data for the intentional and incidental pulls, both of which looked similar despite different behavioral situations.

### Electrophysiological recordings

We obtained multineuronal recordings [39], [41], [43] from individual neurons in the output layer(s) of CFA or RFA while the rats were performing the forelimb movement task or Go/No-go response task. A 16-channel, two-shank or four-shank silicon probe with one or two tetrode-like arrangements in each shank (A2×2-tet-3 mm-150-150-121/312 or A4×1-tet-3 mm-150-121/312; NeuroNexus Technologies, USA) was inserted vertically up to 1,250 µm deep (putative layer 5; cf. Supplementary Fig. 7a of our previous report [39]) into the CFA or RFA, at least one hour before the start of recording experiment. The multichannel signals were amplified with an amplifier (MEG-6116, Nihon Kohden, Japan; or FA-32, Multi Channel Systems, Germany; final gain, 1000 or 2000; band-pass filter, 0.5 Hz to 10 kHz) through a lab-made preamplifier (voltage-follower, gain 1), and digitized at 20 kHz with a 32-channel hard-disc recorder (LX-120, TEAC, Japan). The position of spout-lever was continuously tracked by an angle encoder throughout the behavioral experiments. The EMG activity of the right forelimb was obtained by an amplifier with its head-stage (EX4-400, Dagan, USA; gain, 1000; band-pass filter, 0.3 Hz to 10 kHz) in some experiments.

### Spike activity analysis

Multineuronal recording data were processed offline to isolate spike events by our semiautomatic spike-sorting software, EToS, using wavelet transform and robust variational Bayes procedures [44], [45]. The spike clusters were combined/divided/discarded manually to refine single-neuron clusters by the manual clustering software Klusters and NeuroScope [46]. In this study, we basically focused on the first-order analysis (at a single cell level) to compare the neural activity between the CFA and RFA. In each neuron (spike cluster), its basal spiking properties and functional activity in relation to behavioral task performance were analyzed via MATLAB (The MathWorks) as follows. The ongoing (all averaged) spike rate and spike duration for individual spike clusters were defined in the same manner as described in our previous studies [39], [41]. The spike clusters were then classified into regular-spiking (RS) and fast-spiking (FS) neuron subtypes according to the spike duration (e.g., CFA-RS for the RS subtype in CFA). The coefficient of variation (CV) of spiking activity [specifically, inter-spike interval (ISI)] was calculated by dividing the standard deviation (s.d.) of ISI by the mean of ISI. A temporal feature in its autocorrelogram (ACG) was evaluated by defining ''ACG bias'' as the median value in a time-window from 0 to +100 ms in ACG. The ACG bias toward 0 ms denotes burst-like spiking, while over 50 ms denotes tonic spiking.

To examine the functional activity in relation to behavioral task performance, we aligned spike trains in correct trials (≥40 trials, and total ≥50 spikes during all the trials; unless otherwise mentioned) with the end of push (ranging from −500 to +1,000 ms from this event), the onset of pull (−1,000 to +500 ms), and the onset of cue presentation (−1,000 to +500 ms). To define ''task-related'' activity, the cumulative distribution of all spike positions in the time-course of each trial was compared with that of the same number of uniformly distributed spike positions by using the Kolmogorov-Smirnov (KS) test, where *p*<1×10^−6^ was judged as ''task-related'' in our analysis condition. The small *p* threshold is simply due to the assumption of a uniform distribution of spike positions (instead of shuffled spike positions); we found this criterion is practically more useful and quantitative than our previous criteria [39]. The functional activity was calculated and displayed as Gaussian-filtered spike activity (σ = 100 ms, 0.5 ms bin), which was averaged across all trials and normalized with its peak value.

We defined Hold-type activity as functional activity with its peak in a core of hold period (200–800 ms after the push end, or 200–800 ms before the pull onset). Similarly, Push- and Pull-type activities were defined as functional activities with the peak around the push (−250 to +50 ms from the push end) and pull (−50 to +250 ms from the pull onset), respectively. Pre-pull-type activity was an intermediate form between the Hold- and Pull-type activities (i.e., 50–200 ms before the pull onset). SR_PUSH_, SR_HOLD_, and SR_PULL_ were the averaged spike rates during the time windows for push (−250 to +50 ms from the push end), hold (200–800 ms after the push end, or 200–800 ms before the pull onset), and pull (−50 to +250 ms from the pull onset), respectively. In addition, SR_Go HOLD_ and SR_No-go HOLD_ were the averaged spike rates during the time window for hold (200–800 ms before the intentional/incidental pull onset) in Go and No-go trials, respectively. SR_Go PULL_ and SR_No-go PULL_ were the averaged spike rates during the time window for pull (−50 to +250 ms from the intentional/incidental pull onset) in Go and No-go trials, respectively. The direction preference index (DPI) was defined as (SR_PULL_ – SR_PUSH_)/(SR_PULL_ + SR_PUSH_). If the DPI value is close to 1, the direction preference is considered as ''pull-preferred'', and if 0, it is neutral. Covariance between two neurons in trial-to-trial variability of spike occurrence was analyzed by calculating the correlation coefficient (*r*) of their spike numbers during hold-pull movement (−750 to +250 ms from the pull onset) every trial (200 trials for analysis) [47], [48]. For comparison in population analysis, shuffled data were prepared in each neuron pair by randomizing the original data in blocks of 10 trials to cancel spurious correlation owing to a slow change in spike activity.

### Histological observations

After the recording experiments, the rats were perfused intracardially with cold saline followed by 4% formaldehyde in 0.1 M phosphate buffer under deep anesthesia with urethane (2–3 g/kg, i.p.). Their brains were post-fixed and sliced coronally into 50 µm-thick serial sections by a microslicer (DTK-1500, Dosaka EM, Japan). The sections were mounted on slides, and Nissl-stained with Neutral Red. Electrode tracks were checked in the CFA or RFA of the sections under a microscopy (BX51N, Olympus, Japan).

### Statistics

**Figure 2:**
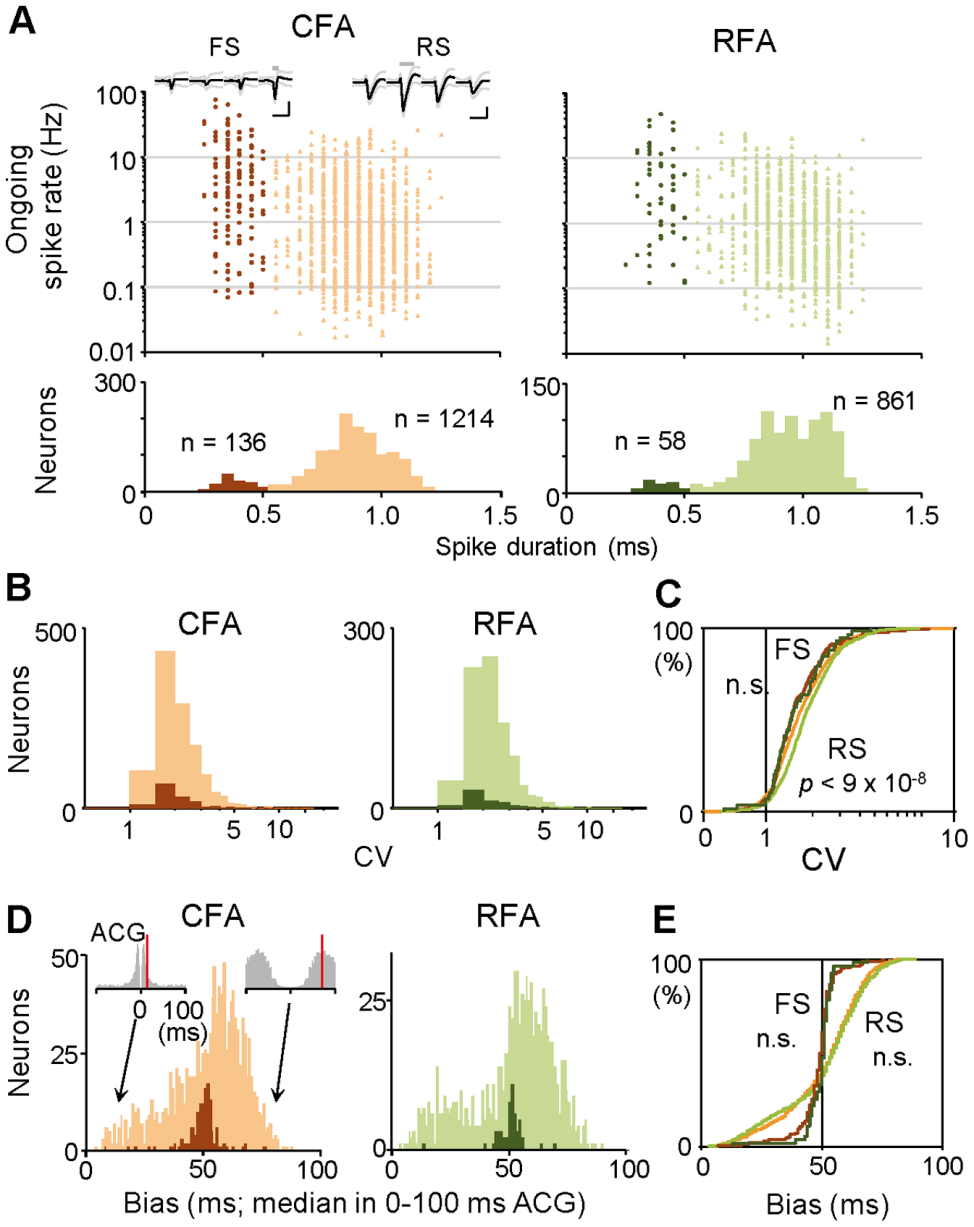
Basal spiking properties of CFA and RFA neurons. **A**) Classification of isolated units in CFA (orange) and RFA (green) into regular-spiking (RS; spike duration >0.5 ms, light colors) and fast-spiking (FS; ≤0.5 ms, dark colors) subtypes of neurons. *Top*: ongoing (all averaged) spike rate plotted against spike duration for individual neurons. *Bottom*: bimodal distribution of spike duration. *Insets*, typical spike waveforms for the two neuron subtypes (mean ± s.d.; calibration: 1 ms, 0.1 mV; gray bar, spike duration). **B**) Coefficient of variation (CV) of inter-spike intervals (ISIs) in CFA and RFA neurons. Histograms show CV distributions for RS (light) and FS (dark) subtypes. **C**) Cumulative probability analysis of the CV distribution shown in **B**. **D**) Temporal feature in auto-correlogram (ACG) in CFA and RFA neurons. We defined ACG bias as a median value in ACG from 0 to +100 ms (red lines in two insets). Histograms show ACG bias distributions for RS (light) and FS (dark) subtypes. **E**) Cumulative probability analysis of the ACG bias distribution shown in **D**.

**Figure 3:**
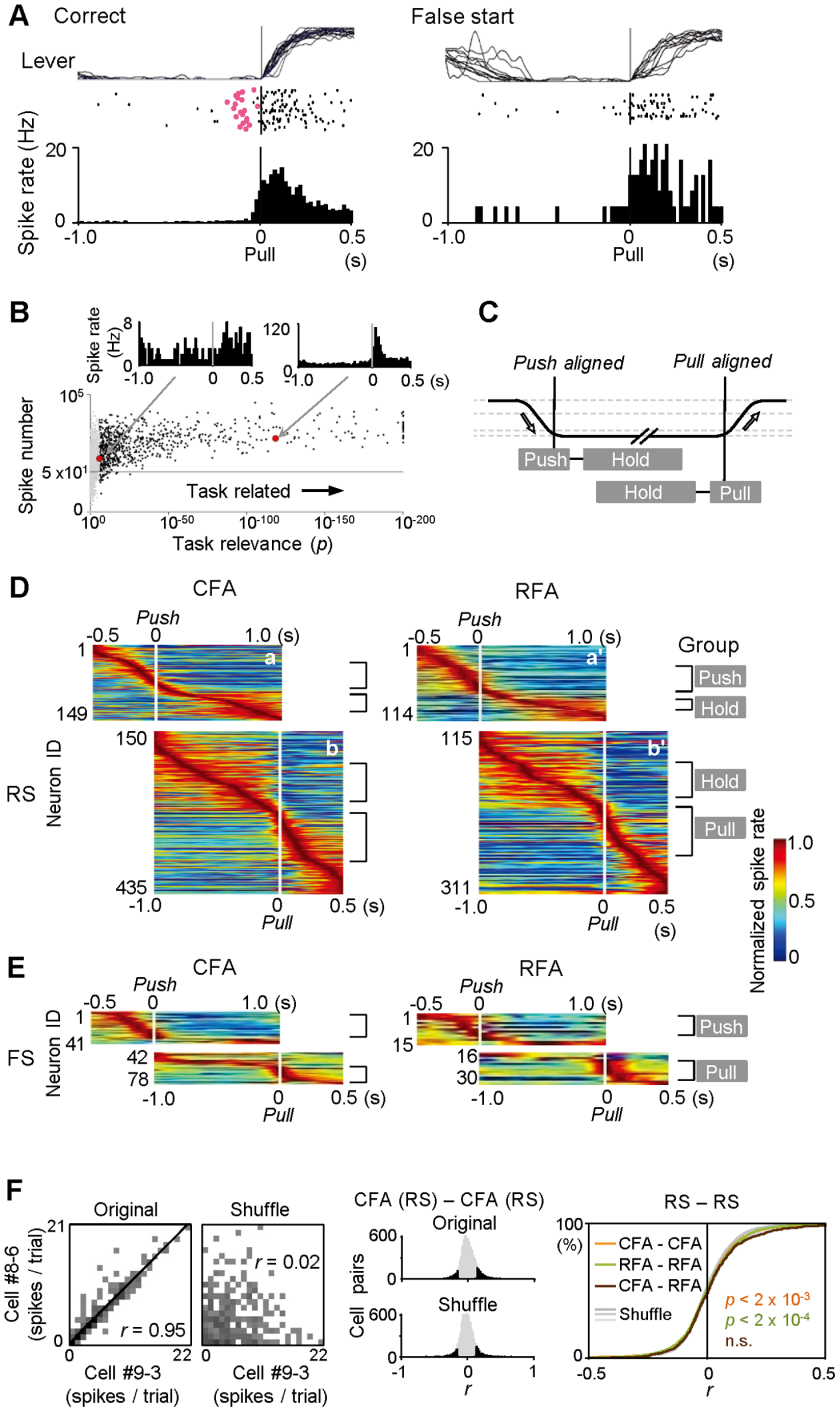
Time-course of functional activity in CFA and RFA neurons. **A**) An example of a neuron (CFA-FS) showing functional (task-related) activity. Spike activity was first aligned with the onset (0 s) of pull movements and then averaged in correct (*left*) and false start (*right*) trials. Black and red dots in raster plots represent spikes and cue onsets, respectively, in consecutive trials (correct, 20 trials; false start, 12 trials). Note the similar activity irrespective of cue presentation. **B**) Definition of task-related activity. The number of spikes during correct trials was plotted against task relevance (*p* in KS test, assuming a uniform distribution) for individual neurons (pull-aligned analysis). Black and gray dots represent the task-related (≥40 trials, ≥50 spikes, and *p*<1×10^−6^) and non-task-related (discarded) neurons, respectively. Insets illustrate two (poorly and well) task-related activities in the plot. **C**) Definition of Hold-, Push-, and Pull-type activities by peak position in push- and pull-aligned analyses. **D**,**E**) Functional activity for RS (**D**) and FS (**E**) subtypes in CFA and RFA. Each row represents normalized Gaussian-filtered spike activity for a single neuron, which was assigned to panel **a** (aligned with the end of push; vertical line at 0 s) or **b** (the onset of pull) according to statistical significance (smaller *p* value). The task-related neurons were sorted by the order of peak time position (early to late). Push-, Hold-, and Pull-type groups are indicated on the right side for further analyses. **F**) Correlation of trial-to-trial variability of spikes between two neurons. *Left*: an example of correlated trial-to-trial spike variability during hold-pull movements in an RS-RS neuron pair (recorded from different electrodes in CFA). *Middle*: populational distribution of correlation coefficient (*r*) in trial-to-trial variability. The *r* distribution was calculated from the original (upper) and shuffled (lower) data (200 trials for analysis) in all pairs of CFA-RS and CFA-RS neurons. Black and gray columns represent neuron pairs with and without statistical significance individually, respectively. *Right*: cumulative *r* distribution in all the pairs of CFA-RS and CFA-RS neurons (orange), of RFA-RS and RFA-RS neurons (green), and of CFA-RS and RFA-RS neurons (brown). Gray lines show distributions from their shuffled data. Note that there were only slight (but significant) differences between the original and shuffled data in the same areas.

Data in the text and figures are expressed as the mean ± s.d. (unless otherwise mentioned) and sample number (*n*). When applicable, we used appropriate statistical tests: i.e., *t*-test (for data analyses in Figs. 2A, 3D (see text), 4B, 5B, 6A,B, and 7A,B), paired *t*-test (Fig. 5B), Kolmogorov-Smirnov (KS) test (Figs. 2C,E, 3B,D-F 4B, 6B, and 7B), two-way ANOVA (Fig. 7C), and *F*-test for s.d. difference (Fig. 7A,B). See Results for details.

## Results

### Behavioral task performance

In the first experiments, a total of 37 rats were trained to perform the forelimb movement task using our task-training system for several days (Fig. 1A) [40]. After task learning, we obtained enough multineuronal recording data from 36 rats for behavioral and electrophysiological analyses. The multineuronal activity was recorded from output layer(s) (putative layer 5) of the CFA and RFA during task performance (Fig. 1A). As expected, the EMG activity in the forelimb was increased during pull/push movements and decreased in the lever-hold period (Fig. 1B). Almost all of the rats successfully learned to perform this behavioral task in which they had to pull the spout-lever quickly in response to the presentation of a cue sound [Fig. 1C; reaction time: mode (peak) 140 ms, ranging from 100 to 640 ms, *N* = 48 sessions from 36 rats].

### Basal spiking properties of CFA and RFA neurons

We cleanly isolated a total of 1,350 CFA neurons and 919 RFA neurons from our multineuronal recordings during task performance. These CFA and RFA neurons were further classified into RS and FS subtypes (e.g., CFA-RS neurons), which should be predominantly excitatory pyramidal cells and inhibitory interneurons, respectively, according to the spike duration (Fig. 2A; it has been validated by our juxtacellular recordings [39]). We examined several spiking properties of all the neurons. First, the ongoing spike rates of CFA-RS neurons were only slightly but significantly higher than those of RFA-RS neurons (Fig. 2A; CFA-RS 2.1±3.5 Hz, *n* = 1,214; RFA-RS 1.7±2.6 Hz, *n* = 861; *t*-test *p*<0.001, which may be an effect of a large sample size), and there was no difference between CFA-FS and RFA-FS neurons (CFA-FS 8.3±12.0 Hz, *n* = 136; RFA-FS 7.0±9.9 Hz, *n* = 58; *t*-test *p*>0.4). Second, the CV of ISI was slightly smaller in CFA-RS than in RFA-RS neurons (Fig. 2B,C; KS test *p*<0.001), and there was no difference between CFA-FS and RFA-FS neurons (*p*>0.9). Third, we also found no populational differences in the temporal feature of ACG (ACG bias) between CFA-RS and RFA-RS neurons (Fig. 2D,E; KS test *p*>0.3) and between CFA-FS and RFA-FS neurons (*p*>0.07). The RS subtype in both areas showed a bimodal distribution in the ACG bias, suggesting that they consisted of burst-like spiking and tonic spiking neurons. Overall, basal spiking properties were essentially very similar in the CFA and RFA neuron subgroups.

### Functional activity during forelimb movements in CFA and RFA neurons

Next, we analyzed functional (task-related) activity in relation to forelimb movements in CFA and RFA neurons. Figure 3A shows a representative neuron that was activated during lever pull movements. The activation started just prior to the onset of lever pull, following the presentation of the cue sound. Importantly, the pull-related activity was observed even in the absence of cue presentation (in false starts); therefore, it appeared to encode mainly motor, rather than sensory (auditory), information, consistent with recording from the motor cortex. We obtained a large number of task-related neurons from the CFA and RFA (Fig. 3B–E; CFA-RS *n* = 435, RFA-RS *n* = 311, CFA-FS *n* = 78, RFA-FS *n* = 30). In particular, those RS neurons were activated at different times in relation to push or pull movements, as reported previously [29], [39]. Many of the RS neurons had a peak of task-related activity during the push or pull movements, while others had peaks in the hold period. The temporal distribution of peak activity was almost the same between CFA-RS and RFA-RS neurons in the push-aligned pooled data (Fig. 3D; KS test *p*>0.8) and in pull-aligned pooled data (*p*>0.5). We also found no difference in the activity earlier than the hold period (in a time-window 1,500 to 1,000 ms before the pull onset) between them [e.g., CFA-RS (Hold-type) 3.2±3.7 Hz, *n* = 102; RFA-RS (Hold-type) 2.7±3.9 Hz, *n* = 59; *t*-test *p*>0.4]. Consistent with our previous study on identified FS interneurons in the CFA [39], most of the CFA-FS and RFA-FS neurons were activated during the push/pull movements, and their temporal patterns were also similar to each other (Fig. 3E; push-aligned, KS test *p*>0.6; pull-aligned, *p*>0.4). Thus, our observation does not support the idea that the RFA (secondary motor cortex) processes hierarchically higher-order motor information as an upstream area than the CFA (primary motor cortex).

To check their functional interactions, we evaluated how trial-to-trial variability of spike activity was correlated between two neurons. There certainly existed a small number of neuron pairs showing significant correlation of trial-to-trial variability (Fig. 3F, *left*). But population analysis using all RS neuron pairs in the same areas revealed that the distribution of correlation coefficient (*r*) was hardly (but significantly) shifted from that using shuffled data (Fig. 3F, *middle* and *right*; *r* among CFA-RS neurons 0.01±0.13, *n* = 7,210, KS test *p*<0.002; *r* among RFA-RS neurons 0.00±0.12, *n* = 5,384, *p*<0.0002; *r* between CFA-RS and RFA-RS neurons 0.03±0.14, *n* = 1,506, *p*>0.8). Such little covariance between two neurons suggests that the animals did not perform improperly multiple forms of pull movements.

**Figure 4:**
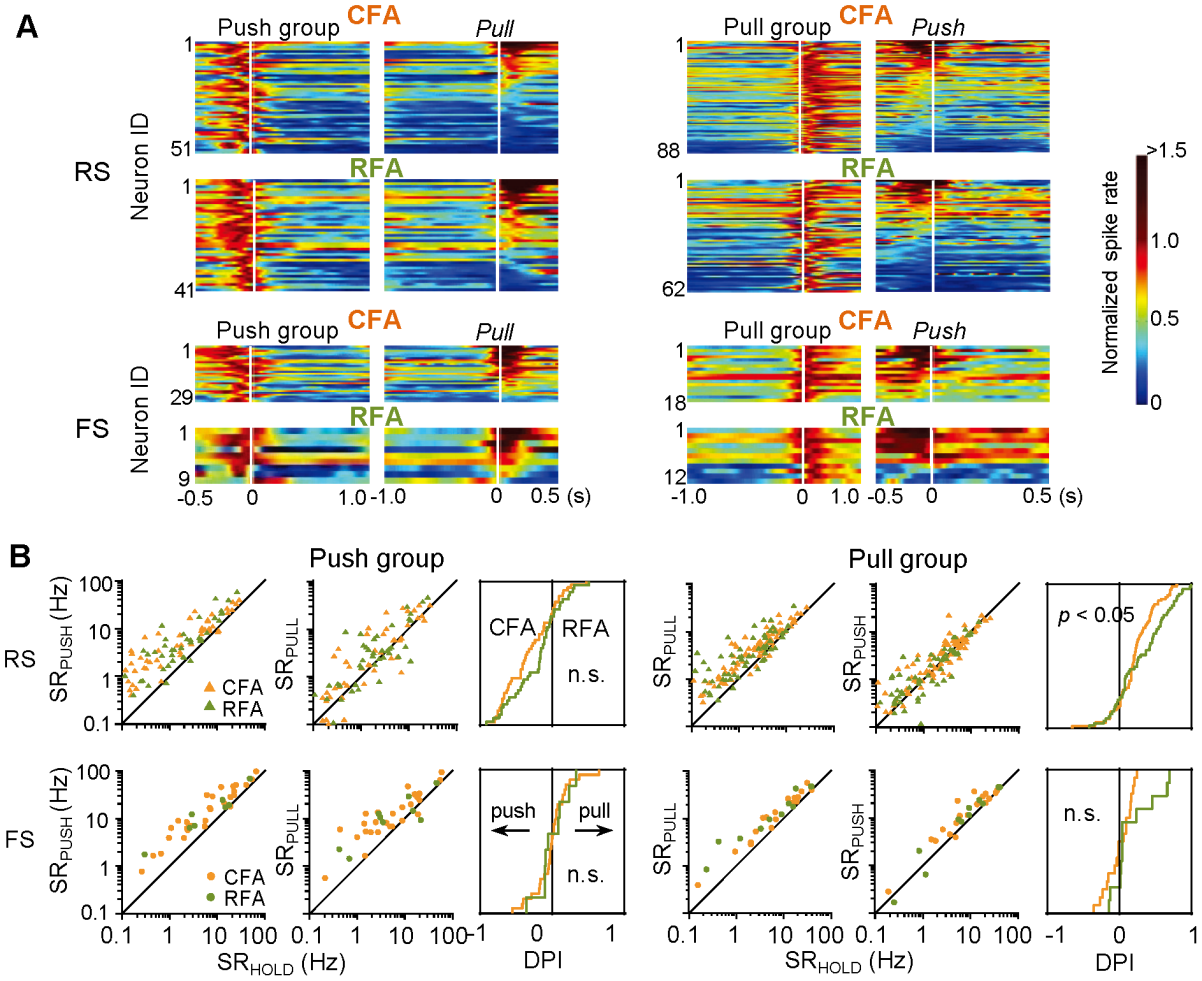
Direction preference of Push-/Pull-type activity in CFA and RFA neurons. **A**) Relative spike rate during forelimb movement in an opposite direction (pull for push, and *visa versa*) in Push- (*left*) and Pull-type (*right*) groups of RS (*upper*) and FS (*lower*) subtypes in each area. In Push-type groups, spike rate was first normalized with the peak activity during push movements in individual neurons, and then, they were sorted by the amplitude of relative spike rate for pull movements (large to small). Pull-type groups were analyzed in a similar way. In this analysis, neurons were included in both Push- and Pull-type groups if they showed significant Push-type activity as well as Pull-type activity. **B**) Spike-rate changes in the Push- and Pull-type groups of RS and FS subtypes (orange, CFA; green, RFA; triangles, RS; circles, FS). Averaged spike rate during push or pull movements (SR_PUSH_, SR_PULL_) was plotted against baseline spike rate in the lever hold period (SR_HOLD_) for individual neurons (*left* and *middle* in each group). Cumulative probability analysis (*right*) shows the distribution of direction preference index [DPI: (SR_PULL_ − SR_PUSH_)/(SR_PULL_ + SR_PUSH_)] in the CFA and RFA neurons.

It is known that individual neurons in the motor cortex often have a preference for one direction of movement [3]. We examined the preferred direction (push or pull) in each neuron that showed a significant peak activity during push and/or pull movements (Push- and Pull-type activity, respectively). As shown in Fig. 4A, about one-third to one-half of the Push-type group of RS neurons in the CFA and RFA exhibited phasic activations in both push and pull directions, and others exhibited no activation or phasic inactivation in the opposite (pull) direction. These Push-type RS neurons showed no populational differences between the CFA and RFA in the spike-rate change from hold to push (Fig. 4B; SR_PUSH_ − SR_HOLD_: CFA-RS 0.42±0.33 in Δlog(spike rate), *n* = 51; RFA-RS 0.32±0.22, *n* = 41; *t*-test *p*>0.07) and from hold to pull (SR_PULL_ − SR_HOLD_: CFA-RS 0.18±0.32; RFA-RS 0.15±0.26; *p*>0.6). Similarly, the Pull-type RS neurons showed no differences between the CFA and RFA in spike-rate changes (SR_PUSH_ − SR_HOLD_: CFA-RS 0.12±0.22, *n* = 62; RFA-RS 0.08±0.24, *n* = 88; *p*>0.4; SR_PULL_ − SR_HOLD_: CFA-RS 0.25±0.19; RFA-RS 0.31±0.27; *p*>0.1). On the other hand, most of the FS neurons in the CFA and RFA exhibited phasic activations in both directions (Fig. 4A), and there were no populational differences between the two areas in spike rate changes (Fig. 4B; for Push-type/Pull-type FS neurons (in this order), SR_PUSH_ − SR_HOLD_: CFA-FS 0.29±0.15/0.16±0.13, *n* = 29/18; RFA-FS 0.34±0.29/0.09±0.13, *n* = 9/12; *p*>0.6/0.1; SR_PULL_ - SR_HOLD_: CFA-FS 0.27±0.21/0.19±0.09; RFA-FS 0.24±0.25/0.25±0.18; *p*>0.7/0.3). Furthermore, the direction preference itself was also similar in the CFA and RFA neurons (Fig. 4B; Push-type RS neurons, CFA-RS −0.31±0.34 in DPI, RFA-RS −0.21±0.34, KS-test, *p*>0.2; Push-type FS neurons, CFA-FS −0.01±0.24, RFA-FS 0.00±0.21, *p*>0.7; Pull-type FS neurons, CFA-FS 0.00±0.18, RFA-FS 0.18±0.30, *p*>0.4), except for one group (Pull-type RS neurons, CFA-RS 0.23±0.27, RFA-RS 0.32±0.35, KS-test *p*<0.05; note it was not significant with a *t*-test, *p*>0.08). These observations suggest that the functional activity of RFA neurons resembles that of CFA neurons in both temporal and spatial aspects of motor information.

**Figure 5:**
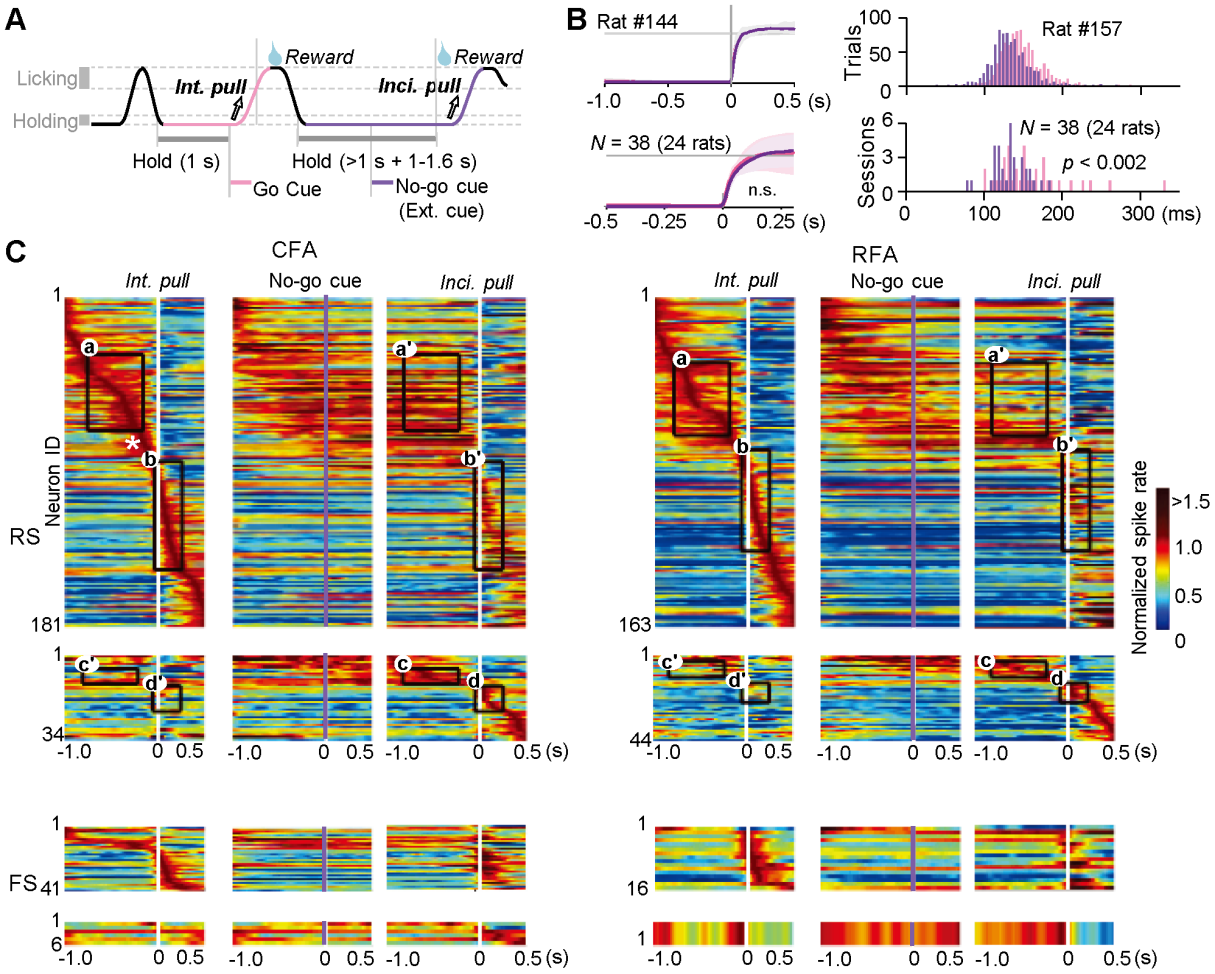
Different modulation of Hold- and Pull-type activities in CFA and RFA neurons. **A**) Intentional (original) and incidental pull movements in a Go/No-go response task. In Go trials, rats must pull the spout-lever deliberately and quickly in response to the presentation of original Go cue to win a reward (Int. pull; 'intentional pull' as a rewarding response). In No-go trials, the rats must keep holding the spout-lever during an extended hold period [1.0–1.6 s after the presentation of No-go (extension) cue]. After the correct No-go response, the rats were allowed to pull the spout-lever to lick the reward anytime (Inci. pull; 'incidental pull' as a consummatory response). Note that the same amount of reward was delivered in both trial types, but more effortful processing would be required for intentional (original) pull movements in the Go trials. **B**) *Left*: averaged lever trajectories (mean ± s.d. traces, aligned with the pull onset) for intentional (pink) and incidental (purple) pull movements in one rat (*top*) and in all of the 38 sessions (24 rats; *bottom*). *Right*: distribution of reaction time for intentional pulls (pink; from Go cue onset to pull onset) and incidental pulls (purple; from reward-pumping noise to pull onset) in one rat (*top*) and all of the rats (*bottom*; latency to peak). **C**) Functional activity aligned with the onset (0 s) of intentional pull in Go trials (1st column from the left), of the No-go cue (2nd) and of incidental pull (3rd) in No-go trials, in RS (*top*) and FS (*bottom*) subtypes of the CFA (*left*) and RFA (*right*) neurons. The spike activity that was significant in the first column (Int. pull-aligned in Go trials) was normalized across the three columns by the peak amplitude from the first column for the individual neurons, which were sorted by the peak time position in the first column (e.g., CFA-RS neurons 1–181). Below, the activity that was significant only in the No-go trials was normalized and sorted by the peak in the third (Inci. pull-aligned) column (e.g., CFA-RS neurons 1–34). Rectangles indicate time windows for Hold- (**a**, **a'**, **c**, **c'**) and Pull-type (**b**, **b'**, **d**, **d'**) activities for comparisons between Go and No-go trials. An asterisk indicates Pre-pull-type activity, which was in between the Hold- and Pull-type activities (see Fig. 6C).

### Different modulation of functional activity between CFA and RFA neurons

Even if CFA and RFA neurons share fundamental motor functions, it is still possible that they play different roles in motor control with major changes in behavioral situation. A large change in behavioral situation would lead to some adaptive changes in internal brain state such as attention, motivation, emotion, and so on. In the second experiments, therefore, we examined whether CFA and RFA neurons differentially encode motor information for similar forelimb movements in two distinct behavioral situations in the Go/No-go response task (Fig. 5A) [40]. In Go trials, rats had to pull the spout-lever as quickly as possible in response to the Go cue presentation for reward acquisition ('intentional pull', a rewarding response); they should conduct auditory cue discrimination and make a decision for the goal-directed action. In No-go trials, they were allowed to pull the lever at will to lick the already-earned reward ('incidental pull', a consummatory response) after a completion of correct No-go response; they should need neither cue discrimination nor decision-making for this action. Operating noise of the micropump for reward delivery (reward-pumping noise) after a correct No-go response could work as another Go signal to allow an incidental pull. The reaction time of intentional pulls (from Go cue onset to pull onset; Fig. 5B, 159.7±47.6 ms) was significantly longer than that of incidental pulls (from reward-pumping noise to pull onset; 129.3±22.1 ms, *t*-test *p*<0.002), suggesting that intentional pulls may require more effortful processing for cue discrimination and decision-making for the goal-directed action, whereas incidental pulls may not require it, but may be facilitated by an attention or motivation to the earned reward. Thus, the behavioral situations were certainly different between the two trial types. In contrast, the rats performed similar pull movements in both trial-types of the Go/No-go response task (Fig. 5B; an example rat: intentional pulls 41.6±3.7% of full shift at 150 ms after the pull onset, *n* = 717 trials; incidental pulls 41.7±3.9%, *n* = 824 trials; paired *t*-test *p*>0.6; group analysis: intentional 40.2±12.2%, incidental 39.3±10.7% at 150 ms after the pull onset; *N* = 38 sessions from 24 rats; paired *t*-test *p*>0.4; see also EMG activity in Fig. 8C of our previous report [40]).

**Figure 6:**
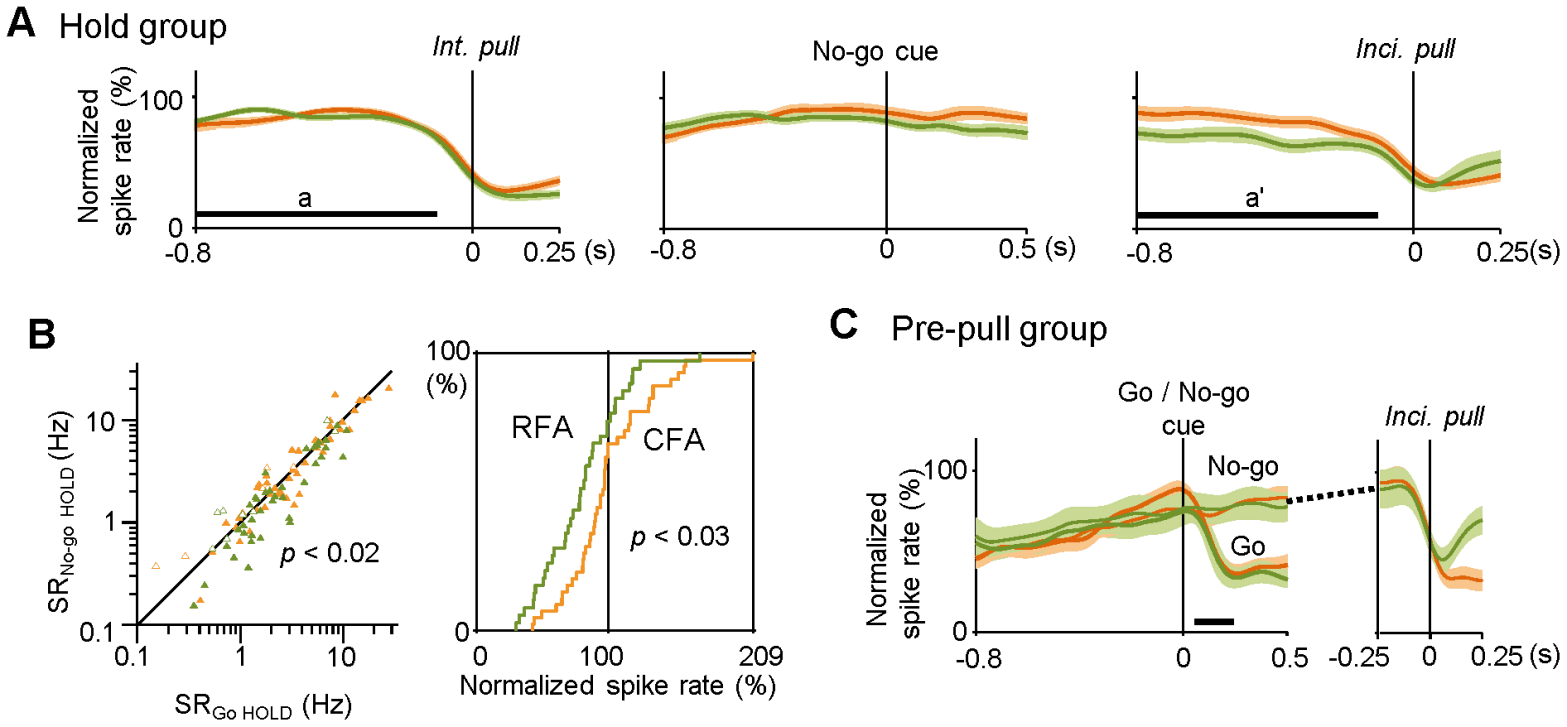
Large reduction in Hold-type activity by an extension of the hold period in RFA-RS neurons. **A**) Populational changes in normalized spike rate in the Hold-type groups (significant in Go trials) of CFA-RS (orange) and RFA-RS (green) neurons [mean ± s.e.m. traces, aligned with the onset (0 s) of intentional pull in Go trials (*left*), No-go cue (*middle*), and incidental pull (*right*) in No-go trials]. Horizontal bars (**a** and **a'**) correspond to the time windows shown in Fig. 5C. Note that the Hold-type activity of RFA-RS was lower than that of CFA-RS neurons in the No-go trials (**a'**), and also that no change was observed in response to the No-go cue presentation. **B**) *Left*: averaged spike rates of Hold-type activity (significant in Go trials) before intentional pull (SR_Go HOLD_, corresponding to Fig. 5C, **a**) and before incidental pull (SR_No-go HOLD_, corresponding to **a'**) for individual CFA-RS (orange, filled triangles) and RFA-RS neurons (green). Open triangles represent those with statistical significance only in No-go trials (corresponding to Fig. 5C, **c'** and **c**). *Right*: cumulative probability analysis of the distribution of normalized spike rates during an extended hold period in No-go trials (**a'**) in CFA-RS and RFA-RS neurons. There was a larger reduction in the Hold-type activity in RFA-RS neurons in the extended period than that in CFA-RS neurons. **C**) Populational changes in normalized spike rate in Pre-pull-type groups of CFA-RS (orange; as indicated by an asterisk in Fig. 5C) and RFA-RS (green) neurons [mean ± s.e.m. traces, aligned with the onset (0 s) of Go or No-go cue (*left*, for Go and No-go trials, respectively) and incidental pull (*right*, for No-go trials)]. A horizontal bar indicates a range of intentional pulls. These types of neurons abruptly stopped a gradually increasing spike activity just prior to intentional/incidental pull movements.

**Figure 7:**
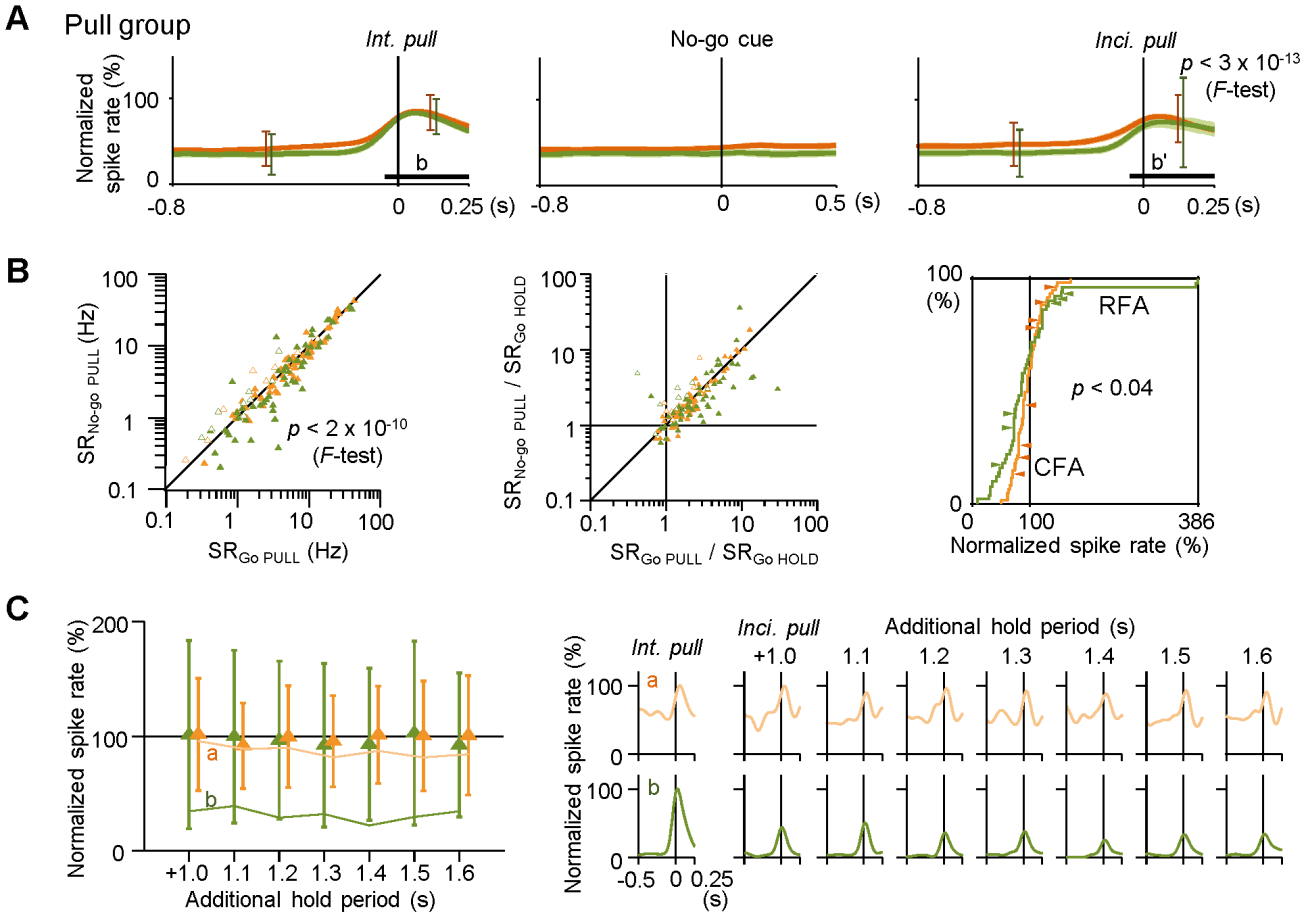
Large amplitude changes in Pull-type activity for intentional and incidental pulls in RFA-RS neurons. **A**) Populational changes in normalized spike rate in the Pull-type groups (significant in Go trials) of CFA-RS (orange) and RFA-RS (green) neurons [mean ± s.e.m. traces, aligned with the onset (0 s) of intentional pull in Go trials (*left*), the No-go cue (*middle*), and incidental pull (*right*) in No-go trials]. Horizontal bars (**b** and **b'**) correspond to the time windows shown in Fig. 5C. Vertical error bars indicate s.d. values for CFA-RS (orange) and RFA-RS (green) neurons. Note that RFA-RS neurons showed a larger s.d. value during incidental pulls than CFA-RS neurons, and also that no change was observed in response to the No-go cue presentation. **B**) *Left*: averaged spike rates of Pull-type activity (significant in Go trials) during intentional pulls (SR_Go PULL_, corresponding to Fig. 5C, **b**) and during incidental pulls (SR_No-go PULL_, corresponding to **b'**) for individual CFA-RS (orange, filled triangles) and RFA-RS neurons (green). Open triangles represent those with statistical significance only in the No-go trials (corresponding to Fig. 5C, **d'** and **d**). *Middle*: relative Pull-type activity that was normalized with the baseline spike rate (SR_Go HOLD_) in the same neurons that are shown in the left. *Right*: cumulative probability analysis of the distribution of normalized spike rates during incidental pulls (**b'**) in the CFA-RS and RFA-RS neurons. The Pull-type activity of RFA-RS neurons was increased or decreased more extensively than that of CFA-RS neurons. Arrowheads indicate representative neurons that were simultaneously recorded from CFA (orange) or from RFA (green). **C**) *Left*: larger Pull-type activity changes were found in the RFA-RS neurons than in the CFA-RS neurons across varying extended hold periods [1.0–1.6 s from the No-go (extension) cue to reward delivery]. *Right*: Pull-type activities in two representative neurons for CFA (a) and RFA (b), indicated by polylines in the left panel.

We obtained a number of task-related CFA and RFA neurons from the 24 rats performing the Go/No-go response task (Fig. 5C; CFA-RS *n* = 215, RFA-RS *n* = 207, CFA-FS *n* = 47, RFA-FS *n* = 17). In this task situation, the No-go cue worked as an extension cue to indicate that lever hold should be extended until the reward was delivered. We failed to find any No-go-cue-specific activity in the RS and FS subtypes of the CFA and RFA (Figs. 5C, 6A, and 7A). In addition, we found no auditory response to the reward-pumping noise in Go or No-go trials (data not shown). Accordingly, these motor cortices seem to have no sensory (auditory) or cognitive function to process the Go/No-go signals in our experimental condition. We, therefore, focused on fundamental motor functions, especially the Hold- and Pull-type activities in RS neurons, with regard to the intentional and incidental pull movements. When the lever hold was extended in No-go trials, the Hold-type activity was prolonged until the incidental pull occurred (Figs. 5C and 6A). The prolonged Hold-type activity (a') was more significantly reduced in the RFA-RS neurons than in the CFA-RS neurons [Fig. 6A, *right*; normalized spike rate in the time window a': CFA-RS 99.7±33.2%, *n* = 43; RFA-RS 79.8±29.9%, *n* = 37; *t*-test *p*<0.005; and similarly, Fig. 6B, *right*, KS test *p*<0.03; Fig. 6B, *left*, SR_No-go HOLD_ − SR_Go HOLD_ (including neurons with significant activity only in No-go trials): CFA-RS −0.00±0.16 in Δlog(spike rate); RFA-RS −0.09±0.19; *t*-test *p*<0.02]. Besides the Hold-type activity, Pre-pull-type activity (e.g., Fig. 5C, asterisk) showed a gradual increase in spike rate until just before the pull onset, regardless of the different behavioral situations (Fig. 6C; CFA-RS *n* = 16, RFA-RS *n* = 7), suggesting that Pre-pull-type activity is associated with motor preparation or initiation.

**Figure 8:**
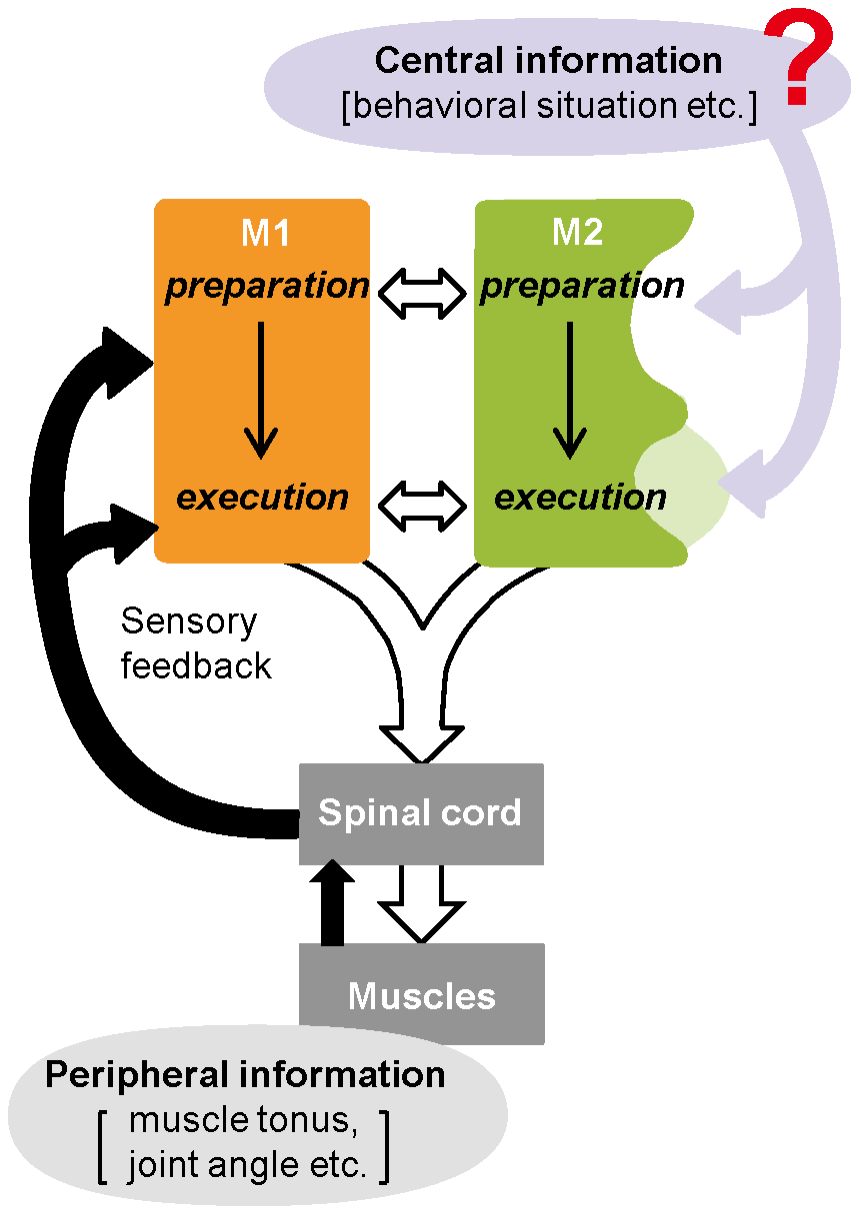
A hypothetical model. Our schematic model of different motor functions of the primary and secondary motor cortices (M1 and M2) in voluntary movement control. See Discussion for details.

On the other hand, the amplitude of Pull-type activity, on average, appeared unaffected between the intentional and incidental pulls in both CFA-RS neurons and RFA-RS neurons (Figs. 5C and 7A; normalized spike rate in the time window b': CFA-RS 95.4±23.6%, *n* = 60; RFA-RS 95.4±67.7%, *n* = 50; *t*-test *p*>0.9). However, the variation (s.d.) of Pull-type activity for incidental pulls was greater in the RFA-RS neurons (i.e., s.d. = 67.7) than in the CFA-RS neurons (23.6) (Fig. 7A, *right*; *F*-test *p*<0.0001); consequently, their cumulative distributions were also significantly different (Fig. 7B, *right*; KS test *p*<0.04). Such different distributions could result from one or two animals with biased neurons (e.g., due to distorted recording site). But it is quite unlikely because there was no biased activity found in simultaneous recordings at two distant sites inside of the same areas (ranging anterior/lateral ±1.0 mm in CFA and ±0.2 mm in RFA; data not shown), and because an unbiased and sparse distribution was observed in most animals individually (Fig. 7B, *right*, arrowheads). Also, this difference was preserved even if outlier points (the highest two points; >3 s.d. from the mean, sampled from different rats) were excluded in the RFA data (KS test *p*<0.02; and also, *F*-test *p*<0.02). This was confirmed by another statistical analysis with actual spike rates [Fig. 7B, *left*; SR_No-go PULL_ − SR_Go PULL_ (including neurons with significant activity only in No-go trials): CFA-RS −0.01±0.13 in Δlog(spike rate); RFA-RS −0.04±0.30; *t*-test *p*>0.4 and *F*-test *p*<0.0001]. Depending on a behavioral situation change, the RFA-RS neurons increased or decreased the Pull-type activity, but neither produced or abolished this activity (Fig. 7B, *middle*; again, significant in *F*-test).

In our task condition, the hold time before the incidental pull was longer than that before the intentional pull. Therefore, it is possible that the variation of Pull-type activity depended on total time of the lever hold. To test this possibility, we analyzed the Pull-type activity in CFA-RS and RFA-RS neurons as a function of an additional (extended) hold period in the No-go trials. As shown in Fig. 7C, the mean values of Pull-type activity of both neuron groups were kept near 100% through the different hold periods (two-way ANOVA, *p*>0.4 for neuron group, *p*>0.5 for hold period), while the s.d. values were always higher in the RFA-RS neurons than in the CFA-RS neurons (*p*<0.001 for neuron group, *p*>0.5 for hold period). This suggests that the variation of Pull-type activity in RFA-RS neurons is dependent on a change in behavioral situation itself, but not on the time of the lever hold.

## Discussion

In the present study, we examined the functional activation of RS and FS neuron subtypes (mostly pyramidal cells and interneurons, respectively [39]) in layer 5 of the CFA and RFA while rats were performing skilled forelimb movements (see Fig. 1) [40]. We showed virtually no major differences between CFA and RFA neurons not only in basal spiking properties but also in the time-course, amplitude, and direction preference of their functional activation for the forelimb movements (Figs. 2–4). On closer inspection, however, we found that the RFA-RS neurons, compared with the CFA-RS neurons, were more susceptible to an alteration in the behavioral situation (Figs. 5–7; rewarding response vs. consummatory response). For instance, the Hold-type activity of RFA-RS neurons was quickly diminished when the lever hold was extended, while the Pull-type activity of RFA-RS neurons was increased or decreased largely. Importantly, CFA and RFA neurons never displayed any No-go-cue-specific activity as a higher-order cognitive/motor function. These observations suggest that the CFA and RFA neurons commonly process fundamental motor information to control skilled forelimb movements, and in addition, that the RFA neurons may be specifically differentiated to modulate motor information with the information of an internal brain state according to rewarding or consummatory response. To our knowledge, this is the first study that clearly shows different modulation of motor information in identified forelimb subfields for the primary and secondary motor cortices in rodents.

It is clear that the activation of CFA neurons elicits skilled and non-skilled forelimb movements in a well-organized manner [39], [41], [49]–[51]. RFA neurons appear to behave similarly to CFA neurons during skilled forelimb movements [23], [29], [30]. Inactivation or disruption of either CFA neurons or RFA neurons severely impairs skilled forelimb movements [20], [23], [34], suggesting their critical contribution to motor control. We now have confirmed that the RFA neuron repertoire is functionally correspondent to the CFA neuron repertoire for simple skilled forelimb movements. Although we could not exclude the possibility that the similarity was merely because of our simple behavioral task or rough data analysis, it seems most likely that the rodent primary and secondary motor cortices, projecting to the spinal cord in parallel [25], [28], are not strictly hierarchical but basically equipollent in the control of voluntary movements (as illustrated in Fig. 8), unlike the primate motor cortices. Alternatively, it is possible that the CFA and RFA neurons control proximal and distal parts of the forelimb, respectively, for the same skilled forelimb movement [14], [22]. In any case, they would communicate to process common motor information through reciprocal cortico–cortical connections directly [25]–[27] and/or through indirect connections via the basal ganglia and thalamus [16], [25], [26], [52], [53]. Although these direct and indirect connections individually originate from and project to different layers of the two motor cortices (e.g. [25]), the functional repertoire of motor cortex neurons, including the Hold- and Pull-type neurons, are distributed across cortical layers (multilayer activation [39], [41]). It is, thus, quite possible that neuron populations in the primary and secondary motor cortices interact with each other through those connections to control voluntary movements cooperatively. Indeed, slow and fast gamma oscillations of neuronal population occur depending on forelimb movements [43] in both the CFA and RFA in a highly coherent manner (Samura et al., unpublished observation).

We found that the Hold- and Pull-type activities of RFA neurons were modulated by a change in internal brain state more extensively than those of CFA neurons. In the RFA neurons, the Hold-type activity, which may engage motor preparation or stillness, was greater before the presentation of the No-go (extension) cue signal than it was after the presentation. Similarly, the Pull-type activity during motor execution depended on the behavioral purpose of pull movements for Go or No-go trials. The No-go cue presentation would probably cease effortful processing such as cue discrimination and decision-making for goal-directed action in our behavioral task condition. This means that these RFA neurons might encode adaptive motor information that is integrated with information of an internal brain state as a result of the effortful processing. Their integrated motor information is advantageous when seeking an optimal motor behavior in an altered internal condition. On the other hand, the functional activation of CFA neurons was less affected by the internal brain state change, which is consistent with our previous study showing that CFA neurons encode no or little reward information [41]. Instead, the CFA neurons received much more somatosensory feedback input than the RFA neurons in awake rats [54], [55]. Taken together, it is likely that the rodent secondary motor cortex neurons may integrate motor information with central information in relation to sensory discrimination, motor decision-making and so on in a top-down manner as an adaptation to a particular behavioral situation (rewarding response or consummatory response), while the primary motor cortex neurons may integrate motor information with peripheral information (somatosensory feedback) in a bottom-up manner for precise control of skeletal muscles (Fig. 8).

Given that the rodent secondary motor cortex is differentiated from the primary motor cortex in terms of motor control function, it is unclear whether the secondary motor cortex has higher-order cognitive/motor functions beyond motor control. For example, when a monkey performs the Go/No-go discrimination task, a group of neurons in the dorsolateral prefrontal cortex show specific cognitive activity in response to a No-go cue signal [56], [57]. Likewise, No-go-related activity is detected in the subareas of the primate motor cortices (SMA [4], [5], PM [8], and CMA [11]), with the exception of the primary motor cortex. In our rat experiments, however, we failed to find obvious No-go-cue-specific activity in the RFA or the CFA, suggesting that No-go information is processed outside of these two areas in the rodents. Besides the No-go response, a pharmacological or surgical lesion of the rodent AGm, which is often used as a synonym for the secondary motor cortex, impairs some higher-order cognitive/motor functions such as conditional response [35], action sequence chunking [36], and value-based action selection [37]. However, the AGl and AGm are extensive zones that are defined cytoarchitecturally, and they are not identical to the primary and secondary motor cortices, respectively; these should be defined functionally. First, the CFA (primary motor cortex) is, in fact, partly overlapped by the somatosensory (hence, not agranular) cortex [23], [49], [54], [58]–[60]. Second, the subfields for body, whiskers, and eye in the primary motor cortex are situated in the AGl, AGm, and cingulate area 1, respectively [38]. Third, it is unclear whether the RFA (secondary motor cortex) is located in the AGl [14], [55] or AGm [26], [30]. Fourth, the AGm has topographic connections with different cortices along its rostral–caudal axis [61]. In addition to the above spatial discrepancy, it is technically difficult to make a pharmacological or surgical lesion restricted to a small target area of the frontal cortex. Accordingly, it is still by no means conclusive that the secondary motor cortex itself participates in higher-order cognitive/motor functions in rodents.

In summary, the rodent primary and secondary motor cortices appear to constitute a dual system of motor cortices to cooperatively control voluntary movements by integrating fundamental motor information with peripheral or central information (Fig. 8). This dual system can reliably execute an appropriate movement according to particular circumstances, and may also facilitate intrinsic or therapeutic restoration of impaired motor function after brain damage [20], [34], [62], [63]. The rodent cerebral cortex seems tiny and primitive compared to the primate cerebral cortex, but rodents can satisfactorily perform skillful and purposeful movements with their digits, paws, and forelimbs, using these motor cortices.
